# A case–control study of quadrivalent human papillomavirus vaccine-associated autoimmune adverse events

**DOI:** 10.1007/s10067-014-2846-1

**Published:** 2014-12-23

**Authors:** David A. Geier, Mark R. Geier

**Affiliations:** Institute of Chronic Illnesses, Inc, 14 Redgate Ct, Silver Spring, MD 20905 USA

**Keywords:** Adverse reaction, Autoimmunity, SLE, Vaccination

## Abstract

GARDASIL (Merck & Co., Inc., Whitehouse Station, NJ, USA) is a quadrivalent human papillomavirus (HPV4) vaccine. An epidemiological study was undertaken to evaluate concerns about the potential for HPV4 vaccination to induce serious autoimmune adverse events (SAAEs). The vaccine adverse event reporting system (VAERS) database was examined for adverse event reports associated with vaccines administered from January 2006 through December 2012 to recipients between 18 and 39 years old with a listed residence in the USA and a specified female gender. It was observed that cases with the SAAE outcomes of gastroenteritis (odds ratio (OR) = 4.6, 95 % confidence interval (CI) = 1.3–18.5), arthritis (OR = 2.5, 95 % CI = 1.4–4.3), systemic lupus erythematosus (OR = 5.3, 95 % CI = 1.5–20.5), vasculitis (OR = 4, 95 % CI = 1.01–16.4), alopecia (OR = 8.3, 95 % CI = 4.5–15.9), or CNS conditions (OR = 1.8, 95 % CI = 1.04–2.9) were significantly more likely than controls to have received HPV4 vaccine (median onset of SAAE symptoms from 6 to 55 days post-HPV4 vaccination). Cases with the outcomes of Guillain-Barre syndrome (OR = 0.75, 95 % CI = 0.42–1.3) or thrombocytopenia (OR = 1.3, 95 % CI = 0.48–3.5) were no more likely than controls to have received HPV4 vaccine. Cases with the general health outcomes of infection (OR = 0.72, 95 % CI = 0.27–1.7), conjunctivitis (OR = 0.88, 95 % CI = 0.29–2.7), or diarrhea (OR = 1.01, 95 % CI = 0.83–1.22) were no more likely than controls to have received HPV4 vaccine. Previous case series of SAAEs and biological plausibility support the observed results. Additional studies should be conducted to further evaluate the potential biological mechanisms involved in HPV4 vaccine-associated SAAEs in animal model systems, and to examine the potential epidemiological relationship between HPV4 vaccine-associated SAAEs in other databases and populations.

## Introduction

GARDASIL (Merck & Co., Inc., Whitehouse Station, NJ, USA) is a quadrivalent human papillomavirus (HPV4) vaccine prepared from the purified virus-like particles (VLPs) of the major capsid (L1) protein of HPV types 6, 11, 16, and 18 [1]. The L1 proteins are produced by separate fermentations in recombinant *Saccharomyces cerevisiae* and self-assembled into VLPs. The fermentation process involves growth of *S. cerevisiae* on chemically defined fermentation media which include vitamins, amino acids, mineral salts, and carbohydrates. The VLPs are released from the yeast cells by cell disruption and purified by a series of chemical and physical methods. The purified VLPs are adsorbed on preformed aluminum-containing adjuvant (amorphous aluminum hydroxyphosphate sulfate). The HPV4 vaccine is a sterile liquid suspension that is prepared by combining the adsorbed VLPs of each HPV type and additional amounts of the aluminum-containing adjuvant and the final purification buffer. HPV4 is a sterile suspension for intramuscular administration. Each 0.5 mL dose contains approximately 20 μg of HPV 6 L1 protein, 40 μg of HPV 11 L1 protein, 40 μg of HPV 16 L1 protein, and 20 μg of HPV 18 L1 protein. Each 0.5 mL dose of the vaccine contains approximately 225 μg of aluminum, 9.56 mg of sodium chloride, 0.78 mg of l-histidine, 50 μg of polysorbate 80, 35 μg of sodium borate, <7 μg yeast protein, and water for injection. The product does not contain a preservative or antibiotics. In June 2006, the US Food and Drug Administration (FDA) approved a regimen of three HPV4 injections given over 6 months to women between the ages of 9 and 26 years of age and the advisory committee on immunization practices (ACIP) subsequently recommended routine HPV4 vaccination for girls who are 11–12 years old [2].

It was previously described that the etiology of autoimmune diseases is still not completely clear but genetic, immunological, hormonal, and environmental factors are considered to be important triggers [3]. Most often, autoimmunity is not followed by clinical symptoms unless an additional event such as an environmental factor favors overt expression. Many environmental factors are known to affect the immune system and may play a role as triggers of the autoimmune mosaic, such as bacterial, viral, and parasitic infections. These are known to induce and exacerbate autoimmune diseases, mainly by the mechanism of molecular mimicry. The question of a connection between vaccination and autoimmune illness has long been debated in the literature and is surrounded by controversy [4], but it was suggested that the same mechanisms that act in infectious invasion of the host apply equally to the host response to vaccination [3]. As recently reviewed [5], the introduction of HPV vaccine was associated with several cases of onset or exacerbations of autoimmune diseases following immunization in the literature and pharmacovigilance databases, triggering concerns about its safety. The purpose of the present study was to conduct an epidemiological study of the vaccine adverse event reporting system (VAERS) to evaluate concerns about the potential for HPV4 vaccination to induce serious autoimmune adverse events (SAAEs).

## Materials and methods

The VAERS is an epidemiological database that has been maintained jointly by the US Centers for Disease Control and Prevention (CDC) and FDA since 1990 as a surveillance tool to evaluate vaccine safety. Specific adverse events following vaccination are required to be reported to this database as mandated by law, but other adverse events that occur following vaccine administration are passively reported to VAERS. The VAERS Working Group of the CDC has previously acknowledged that less than 5 % of the total adverse events reported to VAERS are reported by parents. Specific serious adverse events and deaths reported to VAERS are followed-up by the CDC/FDA. The VAERS Working Group of the CDC and the FDA have repeatedly analyzed and published epidemiologic studies based upon VAERS [6, 7].

The VAERS Working Group notes that VAERS is simple to use, flexible by design, and the data are available in a timely fashion, but it also warns that the potential limitations may include systematic error due to underreporting, erroneous reporting, frequent multiple exposures, multiple outcomes, and lack of precise denominators. In addition, when evaluating data from VAERS, it is important to note that, for any reported event, no cause and effect relationship has been established. VAERS is interested in all potential associations between vaccines and adverse events. Therefore, VAERS collects information on any adverse event following vaccination reported by an individual associating the adverse event with vaccination, be it coincidental or truly caused by a vaccine [6, 7].

### Determining the population at risk

An analysis of the VAERS updated through February 2014 was undertaken using the CDC Wonder online computer interface (http://wonder.cdc.gov/vaers.html) and MedAlerts online computer interface (http://www.medalerts.org/vaersdb/index.php). These portals provide a direct method for independent investigators to rapidly analyze up-to-date data in VAERS. Adverse event reports associated with vaccines administered from January 2006 through December 2012 to recipients between 18 and 39 years old with a listed residence in the USA and a specified female gender were used to identify cases and controls in the present study. Overall, a total of 22,011 adverse event reports in females were examined in the present study, and these adverse event reports were reported to VAERS following administration of HPV4 or any other vaccine(s).

### Determining cases

The SAAE cases were selected from the 22,011 total adverse event reports in females examined in the present study and were defined with outcomes specified as gastroenteritis (VAERS code: 10017888), arthritis (VAERS codes: 10003246 or 10039073), Guillain-Barre syndrome (VAERS code: 10018767), thrombocytopenia (VAERS codes: 10043554 or 10043561), systemic lupus erythematosus (VAERS code: 10042945), vasculitis (VAERS code: 10047115), alopecia (VAERS code: 10001760), and central nervous system (CNS) conditions (VAERS codes: 10028245 or 10012305 or 10030942 or 10028524 or 10028527). In addition, general health outcome cases were selected from the 22,011 total adverse event reports in females examined in the present study and were defined with outcomes specified as infection (VAERS code: 10021789), conjunctivitis (VAERS code: 10010741), and diarrhea (VAERS code: 10012735). Table 1 summarizes the total number of cases for each type of outcome examined in VAERS.

**Table 1:** A summary of various types of cases and controls examined in the present study

Outcome examined (VAERS code)	Number
Serious autoimmune adverse events:	
Gastroenteritis cases (10017888)	12
Controls	21,999
Arthritis (10003246 or 10039073)	56
Controls	21,955
Guillain-Barre syndrome (10018767)	97
Controls	21,914
Thrombocytopenia (10043554 or 10043561)	24
Controls	21,987
Systemic lupus erythematosus (10042945)	13
Controls	21,998
Vasculitis (10047115)	11
Controls	22,000
Alopecia (10001760)	56
Controls	21,955
CNS conditions (10028245 or 10012305 or 10030942 or 10028524 or 10028527)	75
Controls	21,936
General health adverse events:	
Infection (10021789)	39
Controls	21,972
Conjunctivitis (10010741)	19
Controls	21,992
Diarrhea (10012735)	639
Controls	21,372

### Determining controls

The controls were selected from the 22,011 total adverse event reports in females examined in the present study. The controls were selected for each type of case outcome examined by including only those adverse event reports that did not include the specific type of case outcome under study. Table 1 summarizes the total number of controls for each type of case outcome examined in VAERS.

### Determining exposure

Exposure was determined in the present study based upon HPV4 vaccine administration (VAERS code: 1098). It was presumed that adverse event reports that included HPV4 vaccine were exposed and adverse event reports that did not include HPV4 vaccine were unexposed.

### Statistical analyses

The Fisher’s exact test contained in the StatsDirect (version 2.8.0) statistical software package was utilized for statistical analyses, and a two-sided *p* value <0.05 was considered to be statistically significant. The null hypothesis was that there would be no difference in exposure to HPV4 vaccine among cases and controls.

## Results

Table 2 examines among cases with SAAEs and controls the frequency of exposure to HPV4 vaccine administration in the VAERS database. It was observed that cases with the outcomes of gastroenteritis (odds ratio = 4.6), arthritis (odds ratio = 2.5), systemic lupus erythematosus (odds ratio = 5.3), vasculitis (odds ratio = 4), alopecia (odds ratio = 8.3), or CNS conditions (odds ratio = 1.8) were significantly more likely than controls to have received HPV4 vaccine. It was observed that cases with the outcomes of Guillain-Barre syndrome (odds ratio = 0.75) or thrombocytopenia (odds ratio = 1.3) were no more likely than controls to have received HPV4 vaccine.

**Table 2:** A summary of exposure to HPV4 vaccine exposure among SAAE cases and controls

Group examined	Number of cases (%)	Number of controls (%)	Odds ratio (95 % CI)	*p* value^1^
Gastroenteritis				
Exposed	7	5117	4.6 (1.3–18.5)	0.019
Unexposed	5	16,882		
Arthritis				
Exposed	24	5,100	2.5 (1.4-4.3)	0.0018
Unexposed	32	16,855		
Guillain-Barre syndrome				
Exposed	18	5106	0.75 (0.42–1.3)	0.33
Unexposed	79	16,808		
Thrombocytopenia				
Exposed	7	5,117	1.3 (0.48-3.5)	0.47
Unexposed	17	16,870		
Systemic lupus erythematosus				
Exposed	8	5116	5.3 (1.5–20.5)	0.007
Unexposed	5	16,882		
Vasculitis				
Exposed	6	5118	4 (1.01–16.4)	0.049
Unexposed	5	16,882		
Alopecia				
Exposed	40	5084	8.3 (4.5–15.9)	<0.0001
Unexposed	16	16,871		
CNS conditions				
Exposed	26	5098	1.8 (1.04–2.9)	0.033
Unexposed	49	16,838		

^1^The Fisher’s exact test was utilized

Table 3 evaluates among cases with general health outcomes and controls the frequency of HPV4 vaccine administration in the VAERS database. It was observed that cases with the outcomes of infection (odds ratio = 0.72), conjunctivitis (odds ratio = 0.88), or diarrhea (odds ratio = 1.01) were no more likely than controls to have received HPV4 vaccine.

**Table 3:** A summary of exposure to HPV4 vaccine exposure among general health outcomes cases and controls

Group examined	Number of cases (%)	Number of controls (%)	Odds ratio (95 % CI)	*p* value^1^
Infection				
Exposed	7	5117	0.72 (0.27–1.7)	0.57
Unexposed	32	16,855		
Conjunctivitis				
Exposed	4	5120	0.88 (0.29–2.7)	0.99
Unexposed	15	16,872		
Diarrhea				
Exposed	150	4974	1.01 (0.83–1.22)	0.92
Unexposed	489	16,398		

^1^The Fisher’s exact test was utilized

Table 4 examines the seriousness and timing of the SAAEs that were significantly associated with HPV4 vaccine administration. It was observed among the SAAEs examined that vasculitis (33 %), gastroenteritis (14.3 %), and systemic lupus erythematosus (12.5 %) were associated with the highest percentages of life-threatening outcomes. It was also observed among the SAAEs examined that CNS conditions (34.6 %), vasculitis (33 %), and arthritis (25 %) were associated with highest percentages of permanent disabilities. Finally, the median onset of symptoms for the SAAEs examined revealed that vasculitis was associated with closest median onset of symptoms following HPV4 vaccination (6 days), and arthritis was associated with the longest median onset of symptoms following HPV4 vaccination (55 days).

**Table 4:** A summary of SAAEs associated with HPV4 vaccination

Group examined (*n*)	Life threatening (%)^1^	Permanent disability (%)^1^	Median onset of symptoms^2^
Gastroenteritis (7)	1 (14.3)	1 (14.3)	7
Arthritis (24)	1 (4.2)	6 (25)	55
Systemic lupus erythematosus (8)	1 (12.5)	1 (12.5)	19
Vasculitis (6)	2 (33)	2 (33)	6
Alopecia (40)	2 (5)	6 (15)	29.5
CNS conditions (26)	2 (7.7)	9 (34.6)	31

^1^Some outcomes may have more than 1 occurrence in any single event report. If data are grouped by any of these items then the number in the may exceed the total number of unique events and the associated percentage of total unique event reports will exceed 100 % in such cases

^2^Calculated based upon those reports with a specified date of onset of symptoms after vaccination

## Discussion

The present epidemiological study of the VAERS database evaluated the potential relationship between HPV4 vaccine administration and the risk for various types of SAAEs. It was observed that the SAAEs of gastroenteritis, arthritis, systemic lupus erythematosus, vasculitis, alopecia, and CNS conditions were associated with HPV4 vaccine administration, whereas the SAAEs of Guillain-Barre syndrome and thrombocytopenia were not associated with HPV4 vaccine administration. In addition, it was observed that the general health outcomes of infection, conjunctivitis, and diarrhea were not associated with HPV4 vaccine administration. The importance of these findings is that the present study provides epidemiological evidence to support an association between HPV4 vaccine administration and specific SAAEs.

The results obtained are consistent with several previous clinical studies. For example, researchers investigated the association between HPV vaccination and autoimmune manifestations compatible with systemic lupus erythematosus or systemic lupus erythematosus-like disease in six women who presented with such symptoms following HPV vaccination [8]. These investigators reported that in their cases, several common features were observed, such as personal or familial susceptibility to autoimmunity or adverse response to a prior dose of the vaccine, both of which may be associated with a higher risk of post-vaccination autoimmunity. Favorable response to immunosuppressant was observed in all patients, and there was a temporal association between HPV vaccine and the appearance of systemic lupus erythematosus-like conditions. Similarly, other investigators observed a case series of three women that had onset or exacerbation of lupus following HPV immunization [9].

In contrast to the results observed in the present study, Arnheim-Dahlstrom et al. [10] evaluated autoimmune, neurological, and venous thromboembolic adverse events after immunization of adolescent girls with HPV4 in Denmark and Sweden. These investigators examined a cohort of 997,585 girls aged 10–17, among whom 296,826 received a total of 696,420 HPV4 vaccine doses. Unlike the present study that examined adverse event reports to the VAERS database, Arnheim-Dahlstrom et al. examined incident hospital diagnosed autoimmune, neurological, and venous thromboembolic events (53 different outcomes) up to 180 days after each HPV4 vaccine dose, and rate ratios of the outcomes were adjusted for age, country, calendar year, parental country of birth, education, and socioeconomic status, comparing vaccinated and unvaccinated person-time. These investigators observed that the rate ratios for 20 of 23 autoimmune events were not significantly increased. Exposure to HPV4 vaccine was significantly associated with Behcet’s syndrome, Raynaud’s disease, and type 1 diabetes, but these investigators described that each of these three outcomes fulfilled only one of three predefined signal strengthening criteria. In addition, these investigators observed that the rate ratios for five neurological events were not significantly increased, and there was no association between exposure to HPV4 vaccine and venous thromboembolism. It is interesting to note that despite the number of individuals examined by Arnheim-Dahlstrom et al., their study was significantly underpowered to even examine many types of outcomes, and of those outcomes examined, in many cases, relatively few outcomes were observed in the HPV4-vaccinated group. This may potentially be a consequence of the fact that the source for detecting outcomes in the Arnheim-Dahlstrom et al. study was from hospital records. It seems reasonable to hypothesize that many of the conditions examined by Arnheim-Dahlstrom et al. would not necessarily require hospitalization, and worse still, even if the condition eventually might require hospitalization, the Arnheim-Dahlstrom et al. study examined the diagnosis of the outcome in a hospital within the first 181 days after vaccination. As a result, for many of the outcomes examined by Arnheim-Dahlstrom et al. that overlap that with the outcomes examined in the present study, it was observed that there similar potential trends for outcomes in both studies (i.e., odds ratio for vasculitis in VAERS = 4 vs. adjusted rate ratio in Arnheim-Dahlstrom study = 1.55 or odds for systemic lupus erythematosus in VAERS = 5.3 vs. adjusted rate ratio in Arnheim-Dahlstrom study = 1.35), but none of the outcomes were significantly associated with HPV4 vaccination in the Arnheim-Dahlstrom et al. study.

Also in contrast to the present study findings, Chao et al. [11] undertook an observation safety study of HPV4 in 189,629 women who receive one or more doses of HPV4 vaccine between August 2006 and March 2008 for new diagnoses of autoimmune conditions within 180 days following each dose of HPV vaccine. These investigators identified new-onset autoimmune conditions among HPV recipients by electronic medical records, but then the medical records were reviewed by clinicians to confirm the diagnosis and determine the date of onset (only 31–40 % of the cases were confirmed as new onset). It was observed that there was no cluster of disease onset in relations to vaccination timing, dose sequence, or age was found for any autoimmune condition. None of the incidence rate ratios was significantly elevated except for Hashimoto’s disease, but further investigation failed to reveal consistent evidence for a safety signal for autoimmune thyroid conditions. These investigators concluded that no autoimmune safety signal was found in women vaccine with HPV4. Once again, the Chao et al. [11] study, just like the previously discussed Arnheim-Dahlstrom et al. [10] study, was apparently significantly underpowered to find potential autoimmune conditions associated with HPV vaccination, since more than half of the new-onset autoimmune cases identified from electronic medical records were subsequently eliminated from the study following review by clinicians.

### Strengths/limitations

The study design used to evaluate the relationship between exposure and outcome was a significant strength of the present study. The method employed to examine VAERS ensured that the exposures to the various types of vaccines studied occurred prior to the outcomes described in the adverse event reports, since those reporting the adverse outcomes associated the outcomes with the vaccines listed in the adverse event reports.

Another strength of the study was that the VAERS data were collected independently of the study design used in the present study. Among those reporting the adverse event reports examined, it was highly unlikely that any of them could have envisioned methods of analysis used to evaluate the potential relationship between HPV4 vaccine and the adverse events examined.

An additional strength of the present study was the specificity of the types of the SAAEs associated with HPV4 vaccine. Namely, despite the fact that a number of different types of potential SAAEs were examined for the relationship with HPV4 vaccine administration, it was observed that systemic lupus erythematosus or systemic lupus erythematosus-like or associated events were associated with HPV4 vaccine administration. These types of outcomes are biologically plausibly associated with HPV infection and HPV4 vaccine administration.

In addition, in further support of the phenomena observed in the present study, an examination of the VAERS database for adverse events for laboratory findings consistent with systemic lupus erythematosus or systemic lupus erythematosus-like or associated events revealed reports with rheumatoid factor positive (VAERS code: 10039080) or antinuclear antibody positive (VAERS code: 10060055) or antiphospholipid antibodies positive (VAERS code: 10048678) in comparison to controls were significantly more likely to be exposed to HPV4 vaccine than unexposed (odds ratio = 4.8, 95 % confidence interval = 2.7–8.7, *p* < 0.0001). Also, among the SAAEs associated with HPV4 vaccine administration, it was observed, consistent with the biological plausible onset window for SAAEs following vaccination, that the median onset of symptoms ranged between 6 and 55 days post-immunization.

However, the results of the present study may have a number of potential limitations. It is possible the results observed may have occurred from unknown biases or cofounders present in the datasets examined. This seems unlikely because a series of general health outcomes were examined, and all were observed to be exposed to HPV4 vaccine at a similar frequency as controls.

An additional potential limitation of the present study of the VAERS database is that VAERS may have shortcomings, such as underreporting, difficulty in determining causal relationship, and a lack of precise denominators. Nevertheless, as previously described by investigators from the CDC, almost all of these types of shortcomings would apply equally to VAERS reports after vaccines administered to similar populations [12]. The case–control method employed in the present study ensured that all of the adverse event reports examined in VAERS were administered to similar populations (i.e., age and gender), and as a result, examining the relative exposure to HPV4 vaccine among the cases and controls identified from the adverse event reports examined in VAERS should provide accurate relative qualitative and quantitative relationships between differing vaccine exposures and adverse outcomes. Additionally, investigators previously employed a similar case–control study design to the one used in the present study to successfully evaluate the potential relationship between vaccine administration and SAAEs [13].

Another potential limitation of the present study is that the results observed may be the result of statistical chance. However, such a possibility would be unlikely given the limited number of statistical tests performed, the highly significant results observed (most *p* values observed were <0.01), and the consistency in the direction and magnitude of the results observed.

Still, other potential limitations of the present study include the possibilities that some of the individuals in VAERS may have had more subtle adverse events that were not brought to the attention of their healthcare providers, healthcare providers may have misdiagnosed some individuals, or some vaccine exposures may not have been appropriately classified. These limitations, while possibly present in the data examined in the current study, should not have significantly impacted the results observed because it is unclear how differential application would have occurred based upon different exposures among cases and controls. Moreover, misclassification occurring in the data examined would tend to bias any results observed toward the null hypothesis, since such effects would result in individuals being placed in the wrong exposure and/or outcome categories examined, and result in decreased statistical power to determine true potential exposure-outcome relationships.

In addition, another potential limitation of the present study is that other sources of exposure among cases and controls were not evaluated. It is possible that the findings may be the result of other components of the vaccines studied which, in isolation or synergistically, interacted with the HPV4 vaccine examined.

Finally, the current study suffers from the potential limitation that analyses were not conducted to further explore the cumulative dosing effects from HPV4 vaccine administration or to compare HPV vaccine administration in comparison to unvaccinated populations. In future studies, it would be worthwhile to further explore these precise-timing and cumulative-doses phenomena. In addition, it would be valuable to evaluate other adverse events, as well as other covariates such as race, prior medical history, etc., that may further affect the magnitude of the adverse effects found.

## Conclusion

In conclusion, the present study provides epidemiological evidence supporting a significant relationship between HPV4 vaccine administration and SAAEs. The results are consistent with a number of previous case-series of SAAEs observed following HPV4 vaccine administration, and are also consistent with the known biological plausibility of vaccine administration to induce SAAEs in some vaccine recipients. In light of the findings of the present study, we recommend that additional studies be conducted to further evaluate the potential biological mechanisms involved in HPV4 vaccine-associated SAAEs in animal model systems, and to examine the potential epidemiological relationship between HPV4 vaccine-associated SAAEs in other databases and populations.

## References

[1] Stanely M, Lowy DR, Frazer I (2006). Prophylactic vaccines: underlying mechanisms. Vaccine.

[2] Markowitz LE, Dunne EF, Saraiyam M, Lawson HW, Chesson H, Unger ER (2007). Quadrivalent human papillomavirus vaccine: recommendations of the Advisory Committee on Immunization Practices (ACIP). MMWR Recomm Rep.

[3] Molina V, Shoenfeld Y (2005). Infection, vaccines and other environmental triggers of autoimmunity. Autoimmunity.

[4] Shoenfeld Y, Aron-Maor A (2000). Vaccination and autoimmunity-'vaccinosis': a dangerous liaison?. J Autoimmun.

[5] Pellegrino P, Carnovale C, Pozzi M, Antoniazzi S, Perrone V, Salvati D, Gentili M, Brusadelli T, Clementi E, Radice S (2014). On the relationship between human papilloma virus vaccine and autoimmune diseases. Autoimmun Rev.

[6] Singleton JA, Lloyd JC, Mootrey GT, Salive ME, Chen RT (1999). An overview of the vaccine adverse event reporting system (VAERS) as a surveillance system. VAERS Work Group Vaccine.

[7] Geier DA, Geier MR (2004). A review of the vaccine adverse event reporting system database. Expert Opin Pharmacother.

[8] Gatto M, Agmon-Levin N, Soriano A, Manna R, Maoz-Segal R, Kivity S, Doria A, Shoenfeld Y (2013). Human papillomavirus vaccine and systemic lupus erythematosus. Clin Rhematol.

[9] Soldevilla HF, Briones SF, Navarra SV (2012). Systemic lupus erythematosus following HPV immunization or infection?. Lupus.

[10] Arnheim-Dahlstrom L, Pasternak B, Svanstrom H, Sparen P, Hviid A (2013). Autoimmune, neurological, and venous thromboembolic adverse events after immunisation of adolescent girls with quadrivalent human papillomavirus vaccine in Denmark and Sweden: cohort study. BMJ.

[11] Chao C, Klein NP, Velicer CM, Sy LS, Slezak JM, Takhar H, Ackerson B, Cheetham TC, Hansen J, Deosaransingh K, Emery M, Liaw KL, Jacobsen SJ (2012). Surveillance of autoimmune conditions following routine use of quadrivalent human papillomavirus vaccine. J Intern Med.

[12] Chen RT, Rosenthal S (1996). An errant critique that misses the mark. Arch Pediatr Adoles Med.

[13] Geier DA, Geier MR (2005). A case–control study of serious autoimmune adverse events following hepatitis B immunization. Autoimmunity.

